# Supplementary material for: TastepepAI: An artificial intelligence platform for taste peptide de novo design
Source: PLoS Comput Biol. 2025 Oct 16;21(10):e1013602. doi: 10.1371/journal.pcbi.1013602 (PMC12543283; doi:10.1371/journal.pcbi.1013602)
Supplement: S1 File — (ZIP) [file pcbi.1013602.s006.zip › Supplementary Data 2/TaPep53 HPLC.doc]

**HPLC REPORT**

| Sample: | NJP93356 TaPep53 AE-5 | | | Analyzed date: | 2024-9-26 |
| --- | --- | --- | --- | --- | --- |
| Analyst: | WJ | | | Reconstitution: | 100%H_2_O |
| Lot. No.: | NJP93356-1172288 | | | | |
| Column: | 4.6×250mm,Venusil MP C18-5 | | | | |
| Solvent A | A: 0.1% Trifluoroacetic Acid in 100% Acetonitrile | | | | |
| Solvent B | B: 0.1% Trifluoroacetic Acid in 100% Water | | | | |
| Gradient: |  | A | B |  | |
|  | 0.0min | 5% | 95% |  | |
|  | 25.0min | 30% | 70% |  | |
|  | 25.1min | 100% | 0% |  | |
|  | 30.0min | Stop | |  | |
| Volume: | 5μl | | |  | |
| Wavelength: | 220nm | | |  | |
| Flow rate: | 1.0ml/min | | |  | |

─────────────────────────────────────────────────────────────

Rank	Time	Conc. Area	Height

────────────────────────────────────────────────────────────

1	6.606	0.3158	17082	2365

2	8.075	0.3835	20742	3996

3	8.295	95.6291	5172223	584366

4	8.500	1.8352	99257	11827

5	8.883	1.8364	99326	8280

─────────────────────────────────────────────────────────────

Total	100	5408630	610834
